# Enhancing Nonverbal Communication Through Virtual Human Technology: Protocol for a Mixed Methods Study

**DOI:** 10.2196/46601

**Published:** 2023-06-06

**Authors:** Analay Perez, Michael D Fetters, John W Creswell, Mark Scerbo, Frederick W Kron, Richard Gonzalez, Lawrence An, Masahito Jimbo, Predrag Klasnja, Timothy C Guetterman

**Affiliations:** 1 Department of Educational Psychology University of Nebraska-Lincoln Lincoln, NE United States; 2 Department of Family Medicine University of Michigan Ann Arbor, MI United States; 3 Department of Psychology Old Dominion University Norfolk, VA United States; 4 Section of General Internal Medicine Department of Internal Medicine Yale School of Medicine New Haven, CT United States; 5 Department of Psychology University of Michigan Ann Arbor, MI United States; 6 Department of Internal Medicine University of Michigan Ann Arbor, MI United States; 7 Department of Family and Community Medicine University of Illinois College of Medicine Chicago, IL United States; 8 School of Information University of Michigan Ann Arbor, MI United States

**Keywords:** human technology, MPathic-VR, nonverbal communication behavior, patient-provider communication, virtual human

## Abstract

**Background:**

Communication is a critical component of the patient-provider relationship; however, limited research exists on the role of nonverbal communication. Virtual human training is an informatics-based educational strategy that offers various benefits in communication skill training directed at providers. Recent informatics-based interventions aimed at improving communication have mainly focused on verbal communication, yet research is needed to better understand how virtual humans can improve verbal and nonverbal communication and further elucidate the patient-provider dyad.

**Objective:**

The purpose of this study is to enhance a conceptual model that incorporates technology to examine verbal and nonverbal components of communication and develop a nonverbal assessment that will be included in the virtual simulation for further testing.

**Methods:**

This study will consist of a multistage mixed methods design, including convergent and exploratory sequential components. A convergent mixed methods study will be conducted to examine the mediating effects of nonverbal communication. Quantitative (eg, MPathic game scores, Kinect nonverbal data, objective structured clinical examination communication score, and Roter Interaction Analysis System and Facial Action Coding System coding of video) and qualitative data (eg, video recordings of MPathic–virtual reality [VR] interventions and student reflections) will be collected simultaneously. Data will be merged to determine the most crucial components of nonverbal behavior in human-computer interaction. An exploratory sequential design will proceed, consisting of a grounded theory qualitative phase. Using theoretical, purposeful sampling, interviews will be conducted with oncology providers probing intentional nonverbal behaviors. The qualitative findings will aid the development of a nonverbal communication model that will be included in a virtual human. The subsequent quantitative strand will incorporate and validate a new automated nonverbal communication behavior assessment into the virtual human simulation, MPathic-VR, by assessing interrater reliability, code interactions, and dyadic data analysis by comparing Kinect responses (system recorded) to manually scored records for specific nonverbal behaviors. Data will be integrated using building integration to develop the automated nonverbal communication behavior assessment and conduct a quality check of these nonverbal features.

**Results:**

Secondary data from the MPathic-VR randomized controlled trial data set (210 medical students and 840 video recordings of interactions) were analyzed in the first part of this study. Results showed differential experiences by performance in the intervention group. Following the analysis of the convergent design, participants consisting of medical providers (n=30) will be recruited for the qualitative phase of the subsequent exploratory sequential design. We plan to complete data collection by July 2023 to analyze and integrate these findings.

**Conclusions:**

The results from this study contribute to the improvement of patient-provider communication, both verbal and nonverbal, including the dissemination of health information and health outcomes for patients. Further, this research aims to transfer to various topical areas, including medication safety, informed consent processes, patient instructions, and treatment adherence between patients and providers.

**International Registered Report Identifier (IRRID):**

DERR1-10.2196/46601

## Introduction

### Overview

The most important component of the patient-provider encounter is communication [1,2]. This finding has been reinforced by the National Cancer Institute model of patient-centered communication [3]. A recent meta-analysis of randomized controlled trials found a statistically significant effect (Cohen *d*=0.11; *P*=.02) on patient outcomes [4], consistent with other systematic reviews [5,6]. Poor patient-provider communication represents a pressing public health issue associated with increased medical errors and malpractice [1,7-11]. Communication has been shown to be a driver of patient satisfaction and outcomes and is closely connected with patient-centered care [9,10,12]. Collectively, these reports stress the public health importance of health communication and the need for interventions to improve providers’ health communication.

Research on verbal health communication has led to important insights into information dissemination in the patient-provider encounter. However, less is known about nonverbal communication despite its equal importance [4]. Mehrabian and Ferris [13] reported that only 7% of emotional communication is conveyed verbally, 38% is conveyed by voice tone and inflections, and 55% is transferred by facial expressions [1,14,15]. Health issues, such as cancer, evoke fear, anxiety, and uncertainty. Patients are keen on subtle cues communicated both verbally and nonverbally [16]. For example, increased direct clinician body orientation is associated with increased patient satisfaction and understanding [17]. Inconsistency between verbal and nonverbal communication is detrimental to patient-centeredness because patients likely perceive these inconsistencies as disingenuous [1]. Henry et al’s [18] systematic review of nonverbal communication found an association between nonverbal communication (global affect, warmth, negativity, listening, etc) and patient satisfaction. Connection to patient outcomes was less clear but also less researched, potentially due to the lack of a systematic assessment of nonverbal behavior. The authors called for developing consistent and validated measures of nonverbal communication and urged that “studies of facial expression might analyze patients’ and clinicians’ expressions at the dyadic rather than the individual level” with dyadic data analysis [18]. Thus, better assessment of nonverbal communication and application of appropriate dyadic statistics are needed.

Communication interventions directed at providers tend to provide communication skills training in a variety of formats, such as courses, workshops, or training videos [6]. One promising informatics-based educational strategy is virtual human training. Technology-based avatars and virtual humans have been developed to address cognitive tasks [19], such as verbal communication [20,21] and reasoning for diagnosis and therapy [22]. However, this body of literature is relatively nascent, particularly related to health communication. Research on virtual humans to enhance health communication has the potential to train medical students and nurses [23,24], as virtual humans offer a unique advantage by providing an authentic, safe simulated environment to learn with the appropriate level of challenge [25]. Users perceive virtual human interactions as real [26], and learners feel a social presence that can enhance learning and engagement [25,27,28]. After receiving feedback, the professional can immediately practice and implement the feedback, which is crucial to developing communication skills [29]. In contrast to human-standardized patients, virtual humans offer needed training without becoming fatigued. As a result, interactive and engaged virtual human informatics–based interventions demonstrate promise in enhancing communication.

Improving communication skills requires a reliable and valid method to assess them. Many communication assessments focus on verbal communication. These methods involve coding recorded interactions, such as the evidence-based Roter Interaction Analysis System (RIAS) [30], which has 41 categories, including a global affect rating (eg, voice tone) and the use of standardized patients instructors (SPIs) in simulated interactions [31] to evaluate performance. A nonverbal assessment is the Facial Action Coding System (FACS), which delineates observable components of facial movement into action units [32,33]. However, these are typically used for summative assessment of interventions or competence. Virtual human and informatics simulations have the potential to use assessment formatively by assessing behavior, providing automated feedback to the user, and giving further opportunities to practice. Educators often distinguish “assessment for learning” (ie, formative) with “assessment of learning” (ie, summative) because strategic use of formative assessment provides a more tailored, engaging experience with better outcomes [34,35]. Unfortunately, with few exceptions [23,36,37], interventions tend to rely on verbal assessment and standardized patients as a summative assessment only, rather than feeding results into the intervention as a formative assessment.

Standardized patients are widely used but are also costly, fatigue-prone, and have reliability concerns. SPIs have been used in objective structured clinical examinations (OSCEs) to assess verbal and nonverbal communication and provide feedback to learners. SPIs and OSCEs are a gold standard in communication assessment and are used in many interventions, including the MPathic–virtual reality [VR] trial, to assess primary outcomes. Yet, the cost of developing the scenarios, finding and hiring SPIs to conduct 1 OSCE, training to reach interrater reliability, and ongoing maintenance training is high. Published costs were estimated at US $35 per student in the United States in 1994 [38] and more recently in 2011 at US $90 per student in a European study [39]. The personnel time required for 1 OSCE has been reported as 104 hours for supervision, monitoring, logistics, and evaluation [39]. Finally, SPIs are prone to fatigue and excessive mental workload, which limits their ability to correctly identify and report on critical conversational and behavioral cues [40]. Therefore, SPIs are costly and best suited for summative assessment.

These informatics-based interventions to improve communication have primarily focused on verbal aspects. A recent innovation is an automated nonverbal feedback and detection system based on teleconferencing with an SPI, as reported by researchers in an Australian medical school [37]. The researchers demonstrated the utility of automated feedback on nonverbal behavior. In a randomized crossover trial, they found the system useful for enhancing medical communication and a statistically significant improvement over the standard curriculum component based on assessments by an SPI. Mpathic-VR, a virtual human simulation, has also assessed nonverbal communication by providing instructions to the user and by collecting sensor data (unanalyzed to date and a focus of this study) on nonverbal behaviors demonstrated. It also included an assessment of nonverbal behavior as 1 of 4 domains in a follow-up OSCE about 1 week post intervention, which indicates training transfer. It is important to note that communication may vary across in-person and telehealth visits. For example, clinicians tend to display more dominant behaviors, fewer empathy utterances, and portray an increased sense of urgency in telehealth visits [41].

One area that remains unclear is how nonverbal behavior is a mediating factor in communication competence, the accuracy of this nonverbal assessment, and whether the assessment is patient-centered. As a mediating factor, a potential path is that the system provides instruction on nonverbal communication (ie, stimulus), which prompts the learners to follow those instructions, leading to improved nonverbal communication as assessed by the Mpathic-VR and the follow-up OSCE. A path model is needed to test these assumptions, beginning with the extent to which the learner followed instructions. Although the data exist in this data set to test that path model, it has not yet been investigated. Understanding the mediating influence of nonverbal communication is essential to fully integrate nonverbal aspects into virtual human technology.

### Conceptual Frameworks

This study is guided by several conceptual frameworks, including frameworks for analysis of human factors and virtual human intervention.

#### Conceptual Framework for Analysis of Human Factors

**Figure 1:**
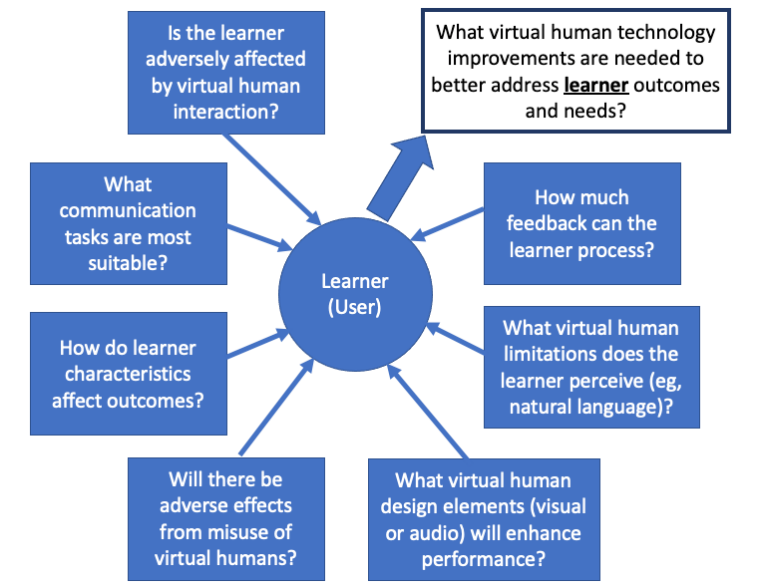
Human factors in virtual human environment. Adapted from Stanney et al [42].

Two models help guide the analysis of the human-computer interaction with the virtual human and the understanding of the mediation of nonverbal behavior. Stanney et al’s [42] human factors in virtual environments (Figure 1) provide a model for systematic informatics research to harness the potential of virtual environments [42]. Second, the Activity Theory–based Model of Serious Games posits 3 interrelated activities contribute—instructional (what MPathic intrinsically teaches), learning (communication outcomes), and gaming (interacting with MPathic) [43]. This model is a framework for analyzing the relationship between the virtual human and educational goals. Teaching communication principles is not enough, and MPathic incorporates audit and feedback principles to reinforce lessons and promote sustained change [44]. A major outcome of this study is to better understand computer-human interaction to ensure a realistic experience that drives behavior change.

#### Conceptual Framework for the Virtual Human Intervention

**Figure 2:**
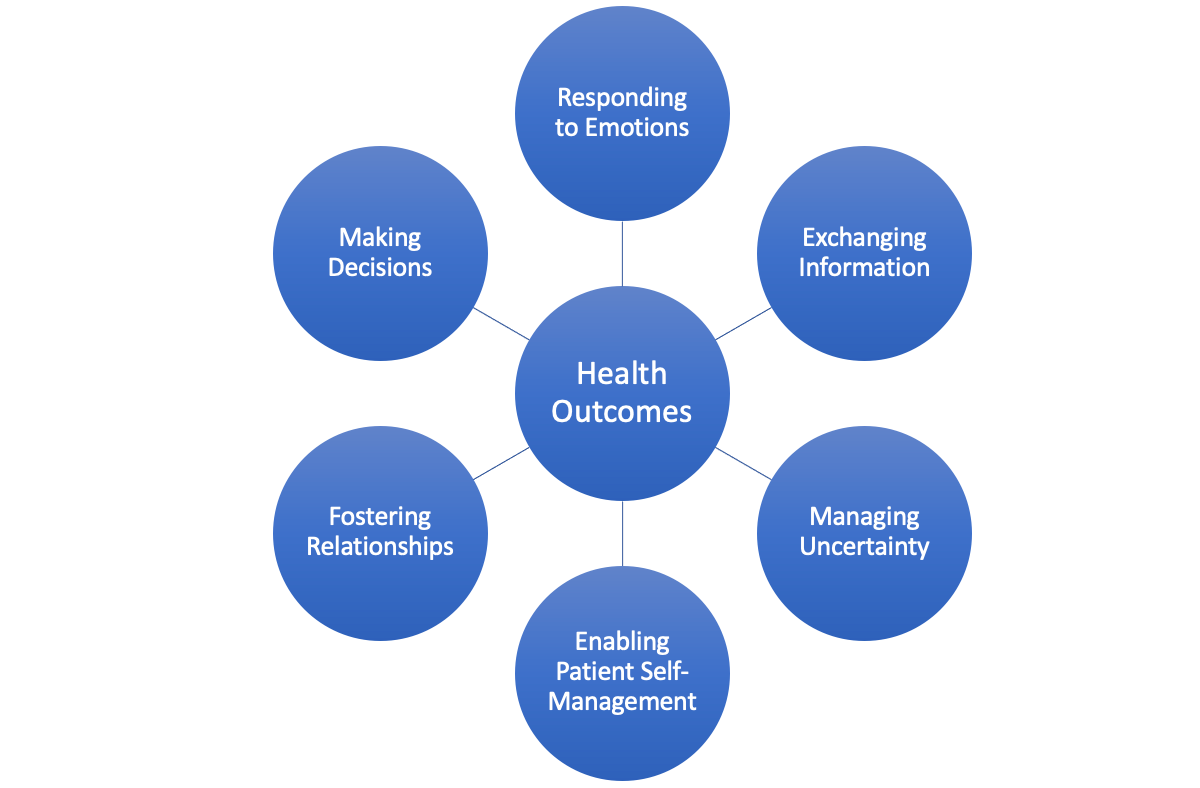
Conceptual model of core functions of patient-clinician communication. Adapted from Epstein and Street [3].

Previous work has developed broad models of patient-centered communication (Figure 2) that guide the virtual human’s instructional tasks. The National Cancer Institute’s model of Patient-Centered Communication in Cancer Care [3] posits that patient-centered care and clinicians skilled in communication lead to improved health communication, which leads to improved health outcomes. Effective communication requires that patients and clinicians have “knowledge, understanding, and self-awareness of what is required to communicate effectively” and the skills to do so. In the Patient-Centered Communication model and consistent with educational theory [45], motivation of all parties is necessary to enhance communication skills. Additionally, health outcomes improve with attention to 6 core communication functions: (1) responding to emotions, (2) exchanging information, (3) making decisions, (4) fostering healing relationships, (5) enabling patient self-management, and (6) managing uncertainty. Nonverbal behavior can be mapped onto these core functions. An intervention that targets these core functions—both verbal and nonverbal aspects—to develop skills and attend to motivation through an engaging learning experience may overcome the limitations of these training models.

### Study Aims

This research aims to improve providers’ dissemination of health information and related health outcomes for patients through an enhanced conceptual model of patient-centered communication that not only describes core communication functions but also presents a directional model of the interrelationships between those components. That conceptual model will be critical to incorporate into the technology to address verbal and nonverbal communication more comprehensively. Finally, this research will build the nonverbal assessment and automated feedback directly into the virtual human simulation for testing. This research has implications for a wide range of disease areas and broad applicability to medication safety, informed consent processes, patient instructions, and treatment adherence.

## Methods

### Overview

This research will use a multistage mixed methods design that includes convergent and exploratory sequential components (Table 1). The first phase will use a convergent design that merges [47] qualitative and quantitative MPathic-VR data to better understand the mediating influence of nonverbal communication (aim 1). Next, an exploratory sequential design will follow that begins with a qualitative grounded theory exploration through patient interviews. This information will be used to build [47] an automated nonverbal communication behavior assessment (aim 2). These findings will be used to develop the virtual human software’s nonverbal assessment and conduct a quality control check as a small pilot of the enhanced system (aim 3).

**Table 1:** Summary of research design: data sources, analysis, and outcomes of each phase.

Aim	Data collection procedures	Data analysis procedures	Product or outcome
(1) Mediating effects nonverbal communication	Quantitative: MPathic game scores, Kinect nonverbal data, OSCE^a^ communication score, RIAS^b^ and FACS^c^ coding of video Qualitative: video recording of MPathic-VR interactions, student reflections	Dyadic data analysis, path modeling Thematic analysis of video data, followed by data transformation to quantify it	Understand what elements of the nonverbal behavior construct are most important in the human-computer interaction
(2) Develop model of nonverbal communication	Interviews with oncology providers about intentional nonverbal behaviors (eg, mirroring, silence, and nodding)	Grounded theory qualitative analysis: open coding and relating components to develop the model	Model of nonverbal communication; function to include in virtual human
(3) Add and validate a new automated nonverbal communication behavior assessment in MPathic-VR	Qualitative findings inform development of assessment along with FACS and RIAS models; Providers use MPathic-VR and assess if the system detects nonverbal behavior	Mixed methods to build assessment: codes → variables, quotes → item language, interrater reliability analysis; code interactions, dyadic data analysis	Updated virtual human with new automated nonverbal communication behavior assessment; initial quality control data

^a^OSCE: objective structured clinical examination.

^b^RIAS: Roter Interaction Analysis System.

^c^FACS: Facial Action Coding System.

### Convergent Mixed Methods Design

#### Overview

A mixed methods convergent design [48] will be used that integrates qualitative nonverbal assessment and student reflections with quantitative MPathic-VR game data and OSCE communication skill scores. The results generated from the convergent mixed methods design will help identify the mediating influence of nonverbal behavior and what nonverbal elements of the intervention were most important.

#### Data Collection

Data will be collected from the MPathic-VR to investigate the mediating influence of nonverbal behavior in virtual human intervention. Although results from the trial indicated a positive effect, it is imperative to understand what aspects of nonverbal communication were most predictive of outcomes. To address this, a large amount of data will be gathered from 210 participants in the MPathic-VR intervention arm (that has not yet been previously analyzed) to understand the extent and mechanism by which nonverbal communication influenced outcomes. Data include video recording MP4 files for each of 4 interactions (840 videos), MPathic-VR scores (continuous data) that reflect the path through the system and responses for each participant, a warehouse of nonverbal sensor data (binary data) recorded by the Microsoft Kinect sensor for 4 nonverbal behaviors (eg, nodding, shaking head, smiles, and proximity), OSCE performance scores (5-point rating for 4 domains and a continuous global score), and qualitative written reflections from the medical students.

#### Data Analysis and Integration

Data will be analyzed using several methods. Specifically, instances of nonverbal behavior displayed by the learner and the virtual human will be coded, and dyadic data analyses will be carried out to examine the extent to which the learner mirrored the behavior of the virtual humans. Then, interactions using the RIAS and FACS will be coded. Another aspect that will be investigated is how participants responded to each virtual human after their action review by using the feedback in their second attempt at the scenario. Thus, it is necessary to test the relationship between the nonverbal behavior the learner demonstrated, and the assessment scores focused on that behavior (ie, if a learner demonstrates nonverbal behavior through the scenario, do the assessments detect it?). These will be tested in a structural equation model that will be compared to the qualitative data.

Importantly, analyzing video and individual-level data will help explain the extent to which participants followed instructions regarding nonverbal behavior and connect it to communication skills outcomes. The R-squared of nonverbal behavior relative to verbal behavior by learners on the OSCE outcome will be calculated to guide virtual human simulations. Data will be integrated by merging the qualitative and quantitative data, which will yield new insight into the particular mechanisms of nonverbal behaviors related to OSCE outcomes. Integration aims to generate meta-inferences, that is, integrated conclusions, beyond what either database alone could determine. The outcomes will provide a better understanding of human-computer interaction with virtual humans, which will inform future informatics interventions and identification of variables to assess nonverbal communication for inclusion in the virtual human.

### Exploratory Sequential Design

#### Overview

To develop a new conceptual model of nonverbal communication to inform virtual human-based training (aim 2) and develop new nonverbal functionality into the MPathic-VR virtual human simulation by creating an automated nonverbal communication behavior assessment for health care providers (aim 3), an exploratory sequential design will be carried out. First, a qualitative study using grounded theory methods [49] will be conducted to explore patient-provider communication, in addition to drawing from existing models. The follow-up quantitative strand will assess how well the system detects added features of nonverbal behavior through a quality control check.

#### Data Collection

For the qualitative grounded theory phase of this exploratory sequential design, data sources will include findings and video data from the MPathic-VR trial (aim 1). Interviews will also be conducted with providers focused on understanding intentional nonverbal communication (eg, mirroring, silence, distance, smiling, nodding, and leaning). Specifically, using theoretical, purposeful sampling [49] of providers (eg, physicians, nurse practitioners, and physician assistants) actively seeing oncology patients, 20 providers will be recruited from the University of Michigan health system, seeking variation in terms of cancer specialty, years since training, gender, and other demographics. The ultimate sample size will be determined by data saturation—the point at which the theory is developed, and new information is not emerging [50]. It is necessary to obtain the providers’ perspectives firsthand of intentional nonverbal communication to develop a patient-centered model. The quantitative strand will consist of video recordings that will allow for comparisons of nonverbal behaviors from provider participants between the system-recorded (Kinect) responses and manually scored records.

#### Data Analysis and Integration

Patient-provider interviews will be analyzed following Corbin and Strauss’ [49] constant comparative method, including open and focused coding, to develop a theory of the process of patient-provider nonverbal communication. This model will provide a theoretical basis for the subsequent automated nonverbal communication behavior assessment I will develop. The outcomes will be a new model of nonverbal communication that refines models of patient-centered communication and novel nonverbal elements to include in MPathic-VR for testing.

The qualitative data analysis, including coding of nonverbal behavior, provider interviews, and the FACS and RIAS models of communication assessment, will inform the development of a novel automated nonverbal communication behavior assessment that will be tested. Specifically, qualitative findings will be incorporated using building integration to systematically use the qualitative findings to develop the assessment. Themes will inform assessment features, and qualitative codes will inform variables to measure in the automated nonverbal communication behavior assessment [51]. The video recordings will be used to assess the reliability of the assessment by calculating Cronbach α using .8 as a standard for internal consistency and will be compared with OSCE nonverbal behavior ratings to gather initial evidence of construct validity. Finally, the manually coded video data will be compared to the Kinect sensor data, both dichotomously scored for each encounter, to gather further evidence of the validity of the assessment. As described above, this existing nonverbal data recorded whether or not 4 nonverbal behaviors (eg, smiles, nods, body lean, and eyebrow raises) occurred for each participant interaction. The new automated nonverbal communication behavior assessment will be programed into the MPathic-VR intervention for quality testing.

A prospective quality check will be conducted to test and refine the new automated nonverbal communication behavior assessment of the MPathic-VR’s ability to capture and assess nonverbal communication using the newly added features. This testing is needed to ensure the virtual human software is assessing clinically meaningful nonverbal behavior. Especially, it will be critical to test whether the system can recognize predefined aspects of nonverbal behavior by comparing system-recorded (Kinect) responses to manually scored records for predefined nonverbal behavior produced by provider test participants (n=30).

All data will be dichotomous (Yes/No) for each nonverbal behavior. Participants will be directed in advance to produce certain behaviors during the scenarios. Then, 2 analysts (principal investigator and research assistant) blinded to the directions will code behavior for each interaction from a video recording using the nonverbal communication behavior assessment, the RIAS, and the FACS. Because the manual recording will be considered the standard, the principal investigator and second independent reader will score manually to account for and investigate variability in manual coding. An interrater reliability analysis of the system-recorded responses with manually scored responses will be conducted. Dyadic data analysis techniques will also be applied to examine recorded nonverbal behaviors.

Based on accepted interrater reliability of SPIs [52,53], κ=0.6 will be considered adequate interrater reliability and κ=0.8 optimal using Cohen statistic [54,55]. Using the most conservative estimate of detecting κ=0.6 and a 2-tailed test of the null hypothesis of 0 agreement, a sample of 30 is needed for 90% power to assess the reliability [55]. If reliability falls below κ=0.6, we have 2 alternative procedures: (1) identify the lowest-performing cue and delete it to improve reliability, or (2) when 1 cue performs poorly, seek new technology, and repeat the analysis with the new technology, and compare to manually-coded assessment data from the MP4 recording. The outcome of this aim will be a refined virtual human software that includes the automated nonverbal communication assessment and initial quality control check data. Aim 3 will be critical to prepare for a subsequent R01 intervention to refine and test the revised virtual human system that contains novel scenarios assessing and automating both nonverbal and verbal communication after action review feedback.

### Ethical Considerations

This study has been approved by the University of Michigan’s institutional review board (HUM00134766). Informed consent will be requested electronically from each participant interested in participating in the study.

### Privacy and Security

Given the large amount of video data that will be used in this study, several steps will be taken to ensure the security of these documents. For example, all data will be stored on University-approved Box and Dropbox secure storage systems using password-protected security and encrypted computers.

## Results

Data collection for this study started in 2019 by accessing secondary data of the MPathic-VR randomized controlled trial data set (210 medical students and 840 video-recordings of interactions) [56]. These data have been analyzed and are expected to be published in 2023. After analyzing these data, recruitment for the qualitative phase of the exploratory sequential design followed in 2021. We expect to complete data analysis for the qualitative phase by July 2023.

## Discussion

### Principal Findings

This study aims to improve providers’ dissemination of health information and related health outcomes for patients through an enhanced conceptual model of patient-centered communication. This study will not only describe core communication functions but also present a directional model of the interrelationships between those components. This conceptual model will be critical to incorporate into the technology to address verbal and nonverbal communication more comprehensively. It is expected that the resulting conceptual model will be able to capture differences in verbal and nonverbal communication behaviors based on the delivery format (eg, in-person or telehealth visits). Finally, the project will build the nonverbal assessment and automated feedback directly into the virtual human simulation for testing. In doing so, we anticipate learners will be able to incorporate the program feedback and improve their nonverbal behaviors.

We expect to find changes across learners interacting with virtual humans on nonverbal behaviors, including those emphasized by the MPathic-VR (eg, eyebrow raise, nodding, and smiling), as well as other facial expressions (eg, furrowed eyebrows, lip corner puller, lower eyelid raise, and oblique eyebrows slight lip press) [57], and head and body orientation (eg, eye gaze toward virtual human, forward lean, and head tilting). Specifically, we would expect to find an increase in the frequency of nonverbal behaviors across learners after repeated interactions with virtual humans. We also anticipate that feedback from the virtual human will contribute to an increased awareness of learners’ nonverbal behaviors. Dissemination of research findings will occur through conferences and articles.

### Limitations

It is important to consider some limitations of this study. First, a team of researchers will be involved in coding a large number of video recordings to analyze learners’ nonverbal behaviors. As a result, coder drift can occur, and discrepancies across coders can arise. To mitigate potential issues of coder drift, the team will meet regularly to discuss any discrepancies throughout the coding process. Second, the role of culture is critical to the assessment of nonverbal behaviors. Nevertheless, a previous study assessing the effectiveness of the MPathic-VR for teaching intercultural and interprofessional communication among medical students demonstrated that the MPathic-VR helped to increase students’ scores after repeated exposure, thus leading to increased awareness of cultural components [36]. Moreover, previous research found significantly higher scores on cultural conversational verbal and nonverbal behaviors for participants exposed to a virtual human condition compared to using illustrations as guides [58]. Thus, the use of virtual humans has been shown to enhance cultural awareness across interactions.

### Conclusions

This research has implications for a wide range of disease areas and broad applicability to medication safety, informed consent processes, patient instructions, and treatment adherence. One of the main strengths of this study is the potential to advance understanding of key aspects of nonverbal communication that can be used to develop informatics-based interventions and ultimately improve patient-provider communication. Using a multistage mixed methods design consisting of convergent and exploratory sequential components, we will be able to more thoroughly understand the nonverbal elements that lead to skill enhancement, develop a model, and automate nonverbal communication behavior assessment. These results will be needed for further virtual human simulation development and will help advance biomedical informatics.

## Appendix

Multimedia Appendix 1 Peer review report from the National Library of Medicine Special Emphasis Panel - Conflict of Interest and Career Awards (National Institutes of Health, USA).

## Abbreviations

FACS: Facial Action Coding System
OSCE: objective structured clinical examination
RIAS: Roter Interaction Analysis System
SPI: standardized patients instructor

## Data Availability

Deidentified data will be made available to all appropriate requests from interested researchers at the time of results publications.
