# Supplementary material for: Enhancing Nonverbal Communication Through Virtual Human Technology: Protocol for a Mixed Methods Study
Source: JMIR Res Protoc. 2023 Jun 6;12:e46601. doi: 10.2196/46601 (PMC10282909; doi:10.2196/46601)
Supplement: Multimedia Appendix 1 [file resprot_v12i1e46601_app1.pdf]

SUMMARY STATEMENT
(Privileged Communication)

PROGRAM CONTACT:
HUA-CHUAN SIM
301-594-4882
simh@mail.nih.gov

Release Date: **04/09/2018**
Revised Date: **04/10/2018**

Application Number: **1 K01 LM012739-01A1**

Principal Investigator

GUETTERMAN, TIMOTHY

Applicant Organization: UNIVERSITY OF MICHIGAN

Review Group:	**ZLM1 ZH-C (01)**<br>**National Library of Medicine Special Emphasis Panel**<br>**Conflict of Interest and Career Awards**
Meeting Date:	**03/23/2018**
Council:	**MAY 2018**
Requested Start:	**07/01/2018**
RFA/PA:	**PAR16-204**
PCC:	**TCDIHC**

Project Title:	**Enhancing Nonverbal Communication through Technology**
SRG Action:	**Impact Score:13**
Next Steps:	**Visit https://grants.nih.gov/grants/next_steps.htm**
**Human Subjects:**	**30-Human subjects involved - Certified, no SRG concerns**
**Animal Subjects:**	**10-No live vertebrate animals involved for competing appl.**
**Gender:**	**1A-Both genders, scientifically acceptable**
**Minority:**	**1A-Minorities and non-minorities, scientifically acceptable**
**Children:**	**3A-No children included, scientifically acceptable**

Project Year	Direct Costs Requested	Estimated Total Cost
1	134,791	145,574
2	137,335	148,322
3	139,954	151,150
TOTAL	412,080	445,046

ADMINISTRATIVE BUDGET NOTE: The budget shown is the requested budget and has not been adjusted to reflect any recommendations made by reviewers. If an award is planned, the costs will be calculated by Institute grants management staff based on the recommendations outlined below in the COMMITTEE BUDGET RECOMMENDATIONS section.

1K01LM012739-01A1 Guetterman, Timothy

BUDGET RECOMMENDATIONS

RESUME AND SUMMARY OF DISCUSSION:

This resubmitted K01 application is from a tenure-track Assistant Professor at the University of Michigan, who has an unusual background in education psychology and who describes a compelling career development plan in the area of non-verbal health communication research using virtual human technology. The candidate has a solid progression from Research Assistant to Fellow to his current position. His training in research methodology around health-related research and the use of virtual technology (MPathic-VR) is timely in applying its use to healthcare applications. VR can prove particularly useful in training individuals if proven reliable to decrease cost, increase provider awareness around the impact of verbal and non-verbal communication to improve interactions with patients with an expected outcome to improve patient care. The training and career development plan is clearly laid out. The primary mentor is outstanding and is an expert in virtual human technology. The environment is very supportive. The applicant has addressed some minor weaknesses of the prior review, such as by increasing his training in health informatics. Overall, this is an outstanding application from a well-qualified candidate who proposes to improve clinical communication, with a specific focus on non-verbal communication. Thus, the enthusiasm is extremely high.

DESCRIPTION (provided by applicant):

This career development award will enable me to develop the necessary skills to conduct and lead biomedical informatics interventions that address crucial health communications issues throughout healthcare. Poor healthcare communication is a serious public health issue that is linked to medical errors, avoidable patient harm and death, suboptimal health outcomes, and low patient satisfaction (1-11). The scientific aims of this health informatics research are to: 1) investigate the mediating influence of computer based nonverbal communication training using virtual human software—MPathic-VR; 2) refine a conceptual model of nonverbal communication; and 3) develop and test an automated assessment of nonverbal communication in the virtual human software. Virtual humans—intelligent, computer-based conversational agents—enable healthcare professionals to develop communication skills through an engaging and active learning experience: practicing communication behavior; receiving assessment feedback on their cognitive, verbal, and nonverbal behaviors; and then practicing again incorporating feedback. A recently concluded trial with the virtual human software found evidence of improved verbal and nonverbal communication skills relative to a computer-based control module condition. However, previous analyses of virtual human software data did not specifically investigate how the software led to improvement in nonverbal communication skills. Aim 1 of this study is to understand how nonverbal behavior improved by conducting a secondary analysis of virtual human trial data using mixed methods. Because nonverbal communication is an important, but often unaddressed, aspect of communication assessment, Aim 2 will develop a new conceptual model of nonverbal communication through interviews with 20 providers. Based on those results, Aim 3 will add an automated nonverbal communication behavior assessment and feedback mechanism to MPathic-VR and conduct a validity check with 30 medical students. My overall career goal is to become an independent investigator focused on applying computer and information sciences to improve dissemination of biomedical information in health settings, which I will accomplish by achieving four training goals: 1) analyze verbal and nonverbal communication, 2) analyze dyadic data, 3) apply virtual human technologies to communication interventions, and 4) obtain advanced skills in grant writing. The mentoring team is experienced with informatics-based interventions, health communication investigations, and mixed methods research. In addition, the University of Michigan and the Department of Family Medicine offer a world-class research infrastructure with strong support for early career investigators through defined programs and courses. Completing this research and the proposed

training goals is critical to developing a rigorous R01 intervention study. This work is also necessary for developing a novel scenario within the MPathic-VR system that can be tested in a future intervention, and it will provide the knowledge needed to advance interactive training in verbal and nonverbal communication.

PUBLIC HEALTH RELEVANCE:

Patient-provider communication is a major public health issue linked to increase risk for medical errors, poor outcomes, and lower patient satisfaction. This research seeks to develop an automated assessment of nonverbal communication in virtual human software so that nonverbal communication may be incorporated into simulations with the goal of enhancing provider communication skills.

CRITIQUES:

The critiques and criterion scores provided below are given by individual reviewers assigned to the application and are presented “as is”, without significant modification or editing by NLM staff. These individual critiques and criterion scores reflect the opinions of these assigned reviewers, and may or may not reflect the final outcome or the final Impact/Priority Score determined by the whole committee.

CRITIQUE 1

Candidate: 3
Career Development Plan/Career Goals /Plan to Provide Mentoring: 3
Research Plan: 3
Mentor(s), Co-Mentor(s), Consultant(s), Collaborator(s): 2
Environment Commitment to the Candidate: 2

Overall Impact:

The candidate has a solid progression from Research Assistant to Fellow to his current position as Assistant Professor at the University of Michigan in the area of Educational Psychology. His training in research methodology around health-related research and the use of virtual technology is timely in applying its use to healthcare applications. VR can prove particularly useful in training individuals if proven reliable to decrease cost, increase provider awareness around the impact of verbal and non-verbal communication to improve interactions with patients with an expected outcome to improve patient care.

1. Candidate:

STRENGTHS

- The candidate has an established track record for research activities is a good basis for becoming an independent researcher.
- The modest publication record was seen as a weakness in the former review; the candidate provided information around evaluation reports for non-research positions. Since the first submission he has served as primary author for an article and has one pending as second author.

2. Career Development Plan/Career Goals & Objectives/Plan to Provide Mentoring:

STRENGTHS

- Training goals are specifically geared to developing or enhancing skills to bolster those areas necessary for the type of research in which he's interested.
- He has chosen a technology that has many untapped possibilities as related to health care. His career plan will enable him to capitalize on the use of VR in the health care arena while fulfilling his goals.
- His prior mixed methods research background is beneficial for understanding and overcoming barriers to successful implementation of healthcare intervention.

3. Research Plan:

STRENGTHS

- It utilizes MPathic-VR focusing how the development of virtual human technology for further use in biomedical setting, specifically provider/patient interactions.
- This updated version has tighter connections to health informatics and is supported by the literature references (comment from previous critique); the design and methodology appear to have scientific merit.
- A contingency has been included if the kappa is below .6 (comment from previous critique).

WEAKNESSES

- It is not clear if medical students are being filmed in four separate instances, or four cameras on a single instance.

4. Mentor(s), Co-Mentor(s), Consultant(s), Collaborator(s):

STRENGTHS

- The research has qualified primary mentors. The addition of a mentor with strong health informatics expertise addresses a concern of the previous review.
- Advisors at his disposal represent clinicians, as well as experts in VR, communications, and specific research methods. They fill potential gaps in specific areas that may not be met by mentors.
- Mentors and advisors have a diversity in background and institutions providing for broader perspective; U of M, Old Dominion, U of CA, U of NE-Lincoln.

5. Environment and Institutional Commitment to the Candidate:

STRENGTHS

- The environment is such that it should prove to be very supportive of this individual's research.
- Resources to support research related to MPathic-VR are substantial.

Protections for Human Subjects: Acceptable Risks and Adequate Protections

- Submit to IRB for approval; protocol for data and safety monitoring

Inclusion of Women, Minorities and Children:

- Sex/Gender: Distribution justified scientifically
- Race/Ethnicity: Distribution justified scientifically

- For NIH-Defined Phase III trials, Plans for valid design and analysis: Not applicable
- Inclusion/Exclusion of Children under 18: Excluding ages <18; justified scientifically
- Women and minorities included; no children

Resubmission: This is a resubmission of an application. The concerns from the initial review appear to have been addressed.

Training in the Responsible Conduct of Research: Acceptable

Comments on Format: Variety of formats; in-person, podcasts, web-based; Local and national offerings.

Comments on Subject Matter: Bioethics, regularity compliance, human subject's protections, research integrity, conflict of interest, HIPAA, PHI

Comments on Faculty Participation: Has completed U-M training

Comments on Duration: 1 - 56 CH

Comments on Frequency: Bi-monthly to 3-4 years

Resource Sharing Plans: Acceptable

Budget and Period of Support: Recommended budget modifications or possible overlap identified:

- Personnel - Salary for the Research assistant (p.78) and the programmer (p.81, Technology Development) is in included in F1-Materials and supplies instead of B-Other Personnel or F3-Consultant Services.
- Other budget items included in F1-Materials and supplies that should reside elsewhere.
- --Training related costs should be in E-Participant/Trainee Support Costs or F8-Other.
- --Hosting should be F8-Other.
- --Transcription should be F8-Other.
- --Publishing should be F2.
- --Participant Payment should be F8-Other. Shouldn't the amount be in increments of 20 (e.g. $320) since participants are being paid $20/each?

CRITIQUE 2

Candidate: 1
Career Development Plan/Career Goals /Plan to Provide Mentoring: 1
Research Plan: 2
Mentor(s), Co-Mentor(s), Consultant(s), Collaborator(s): 1
Environment Commitment to the Candidate: 1

Overall Impact:

This revised K01 application describes a compelling career development plan for a candidate who has the potential to develop into an outstanding investigator in non-verbal health communication research using virtual human technology. The revisions address minor weakness of an already excellent proposal. Strengths include the unusual background of the candidate in education psychology that is

well suited for the project, an outstanding primary mentor who is an expert in virtual human technology to be used in the project, and a very supportive environment.

1. Candidate:

STRENGTHS

- The candidate is well qualified for this project with a PhD in educational psychology and a master's degree in counselling.
- Since previous submission, candidate has published one paper as a first author and has a second one in press as second author.
- Demonstrates very strong potential to become an independent researcher.

WEAKNESSES

- None noted

2. Career Development Plan/Career Goals & Objectives/Plan to Provide Mentoring:

STRENGTHS

- The candidate's goal is to develop methods using virtual human technology to help improve health-related communication.
- There is clearly an articulated plan for interacting with the mentor team.
- Since previous submission, the candidate has added several informatics courses and a mentor with mHealth informatics expertise to already strong development plan.

WEAKNESSES

- None noted

3. Research Plan:

STRENGTHS

- The candidate proposes to use innovative virtual human technology to provide feedback and training in non-verbal health communication.
- Since last submission, he has added human factors modeling and activity based modeling to strengthen informatics relevance.
- The project is well described, feasible and well suited to the candidate's skills.

WEAKNESSES

- None noted

4. Mentor(s), Co-Mentor(s), Consultant(s), Collaborator(s):

STRENGTHS

- Strong mentor team with members who have complimentary expertise.
- The primary mentor, Dr. Fetters, is well established with a track record of successfully mentoring academic investigators.
- Co-mentors have complimentary expertise in virtual human technology, mixed methods and dyadic methods, and mHealth, and are well suited to mentor the candidate.

WEAKNESSES

- None noted

5. Environment and Institutional Commitment to the Candidate:

STRENGTHS

- The Center for Health Communication and Research at the University of Michigan is uniquely suited to the candidate's project.
- The candidate is a tenure track assistant professor in the department of family practice that is supportive of the candidate.

WEAKNESSES

- None noted

Protections for Human Subjects: Acceptable Risks and Adequate Protections

Data and Safety Monitoring Plan (Applicable for Clinical Trials Only): Not Applicable (No Clinical Trials)

Inclusion of Women, Minorities and Children:

- Sex/Gender: Distribution justified scientifically
- Race/Ethnicity: Distribution justified scientifically
- For NIH-Defined Phase III trials, Plans for valid design and analysis: Not applicable
- Inclusion/Exclusion of Children under 18: Excluding ages <18; justified scientifically

Resubmission: This is a resubmission, and the concerns of the initial reviewers have been addressed.

Training in the Responsible Conduct of Research: Acceptable

Resource Sharing Plans: Acceptable

Budget and Period of Support: Recommend as Requested

CRITIQUE 3

Candidate: 1
Career Development Plan/Career Goals /Plan to Provide Mentoring: 1
Research Plan: 1
Mentor(s), Co-Mentor(s), Consultant(s), Collaborator(s): 1
Environment Commitment to the Candidate: 1

Overall Impact:

The applicant has improved the application significantly, especially the areas of concern that was expressed by the reviewers and the program. He was able to address all concerns adequately, including increasing his training in health informatics.

1. Candidate:

STRENGTHS

- The candidate has a unique set of skills based on his training and experience that makes him an ideal applicant for the project.
- Letters included in the application demonstrate strong support for the applicant's research career.

WEAKNESSES

- None

2. Career Development Plan/Career Goals & Objectives/Plan to Provide Mentoring:

STRENGTHS

- The applicant's plan to pursue a relatively under-studied field that matches his expertise improves his chances to achieve his stated career goals significantly.

WEAKNESSES

- None

3. Research Plan:

STRENGTHS

- The proposed project is scientifically rigorous and the plan is well described.
- The topic is unique and may potentially have high impact, especially considering the approach described.

WEAKNESSES

- None

4. Mentor(s), Co-Mentor(s), Consultant(s), Collaborator(s):

STRENGTHS

- There is a complementary set of mentors and consultants, expertise of which cover all aspects of the project.

WEAKNESSES

- None

5. Environment and Institutional Commitment to the Candidate:

STRENGTHS

- The institution supports the applicant at all levels.

WEAKNESSES

- None

Protections for Human Subjects: Acceptable Risks and Adequate Protections

Data and Safety Monitoring Plan (Applicable for Clinical Trials Only): Acceptable

Inclusion of Women, Minorities and Children:

- Sex/Gender: Distribution justified scientifically
- Race/Ethnicity: Distribution justified scientifically
- For NIH-Defined Phase III trials, Plans for valid design and analysis: Not applicable
- Inclusion/Exclusion of Children under 18: Excluding ages <18; justified scientifically

Resubmission: All concerns have been addressed.

Training in the Responsible Conduct of Research: Acceptable

Resource Sharing Plans: Acceptable

Budget and Period of Support: Recommend as Requested

THE FOLLOWING SECTIONS WERE PREPARED BY THE SCIENTIFIC REVIEW OFFICER TO SUMMARIZE THE OUTCOME OF DISCUSSIONS OF THE REVIEW COMMITTEE, OR REVIEWERS' WRITTEN CRITIQUES, ON THE FOLLOWING ISSUES:

PROTECTION OF HUMAN SUBJECTS: ACCEPTABLE

INCLUSION OF WOMEN PLAN: ACCEPTABLE

INCLUSION OF MINORITIES PLAN: ACCEPTABLE

INCLUSION OF CHILDREN PLAN: ACCEPTABLE

COMMITTEE BUDGET RECOMMENDATIONS: The budget clarifications or modifications were requested.

- Personnel - Salary for the Research assistant (p.78) and the programmer (p.81, Technology Development) is in included in F1-Materials and supplies instead of B-Other Personnel or F3-Consultant Services.
- Other budget items included in F1-Materials and supplies that should reside elsewhere.
- --Training related costs should be in E-Participant/Trainee Support Costs or F8-Other.
- --Hosting should be F8-Other.
- --Transcription should be F8-Other.
- --Publishing should be F2.
- --Participant Payment should be F8-Other. Shouldn't the amount be in increments of 20 (e.g. $320) since participants are being paid $20/each?

Footnotes for 1 K01 LM012739-01A1; PI Name: Guetterman, Timothy

NIH has modified its policy regarding the receipt of resubmissions (amended applications). See Guide Notice NOT-OD-14-074 at http://grants.nih.gov/grants/guide/notice-files/NOT-OD-14-074.html. The impact/priority score is calculated after discussion of an application by

averaging the overall scores (1-9) given by all voting reviewers on the committee and multiplying by 10. The criterion scores are submitted prior to the meeting by the individual reviewers assigned to an application, and are not discussed specifically at the review meeting or calculated into the overall impact score. Some applications also receive a percentile ranking. For details on the review process, see http://grants.nih.gov/grants/peer_review_process.htm#scoring.

MEETING ROSTER

National Library of Medicine Special Emphasis Panel
NATIONAL LIBRARY OF MEDICINE
Conflict of Interest and Career Awards
ZLM1 ZH-C (01)
03/23/2018

Notice of NIH Policy to All Applicants: Meeting rosters are provided for information purposes only. Applicant investigators and institutional officials must not communicate directly with study section members about an application before or after the review. Failure to observe this policy will create a serious breach of integrity in the peer review process, and may lead to actions outlined in NOT-OD-14-073 at https://grants.nih.gov/grants/guide/notice-files/NOT-OD-14-073.html and NOT-OD-15-106 at https://grants.nih.gov/grants/guide/notice-files/NOT-OD-15-106.html, including removal of the application from immediate review.

CHAIRPERSON(S)

LEHMANN, HAROLD P, MD, PHD
PROFESSOR
SCHOOL OF MEDICINE
THE JOHNS HOPKINS UNIVERSITY
BALTIMORE, MD 21287

MEMBERS

BUI, ALEX, PHD
DIRECTOR
MEDICAL IMAGING INFORMATICS (MII) GROUP
UCLA GEFFEN SCHOOL OF MEDICINE
LOS ANGELES, CA 90024

ELKIN, PETER L, MD
PROFESSOR AND CHAIR, DEPARTMENT OF BIOMEDICAL INFORMATICS
JACOBS SCHOOL OF MEDICINE AND BIOMEDICAL SCIENCES, UNIVERSITY OF BUFFALO
STATE UNIVERSITY OF NEW YORK
BUFFALO, NY 14203

LENERT, LESLIE A, MD
PROFESSOR OF INTERNAL MEDICINE
CHIEF RESEARCH INFOMRATION OFFICER
DIRECTOR, BIOMEDICAL INFORMATICS CENTER
MEDICAL UNIVERSITY OF SOUTH CAROLINA
VP AND CMO, HEALTH SCIENCES SOUTH CAROLINA
CHARLESTON, SC 29425

MUNGALL, CHRISTOPHER J, PHD
SCIENTIST
ENVIRONMENTAL GENOMICS AND SYSTEMS
LAWRENCE BERKELEY NATIONAL NATIONAL LABORATORY
BERKELEY, CA 94720

OZAYDIN, BUNYAMIN, PHD
ASSISTANT PROFESSOR, HEALTH INFORMATICS
DEPARTMENT OF HEALTH SERVICES ADMINISTRATION
THE UNIVERSITY OF ALABAMA AT BIRMINGHAM
BIRMINGHAM, AL 35294-1212

PEARSON, WILLIAM R, PHD
PROFESSOR
BIOCHEMISTRY AND MOLECULAR GENETICS
UNIVERSITY OF VIRGINIA
CHARLOTTESVILLE, VA 22908

POLLIN, TONI I, PHD
ASSOCIATE PROFESSOR
MEDICINE AND EPIDEMIOLOGY & PUBLIC HEALTH
UNIVERSITY OF MARYLAND SCHOOL OF MEDICINE
BALTIMORE, MD 21201

SCHLEYER, TITUS K, PHD
RESEARCH SCIENTIST
PROFESSOR OF BIOMEDICAL INFORMATICS
CENTER FOR BIOMEDICAL INFORMATICS
REGENSTRIEF INSTITUTE
INDIANAPOLIS, IN 46202

SHOJAIE, ALI, PHD
ASSOCIATE PROFESSOR OF BIOSTATISTICS
DEPARTMENT OF BIOSTATISTICS
UNIVERSITY OF WASHINGTON
SEATTLE, WA 92521

SUMMERS-ABLES, JOY E, MLS
DIRECTOR
ROBERT M. BIRD HEALTH SCIENCES LIBRARY
UNIVERSITY OF OKLAHOMA HEALTH SCIENCES LIBRARY
OKLAHOMA CITY, OK 73126

VEINOT, TIFFANY CE, PHD
ASSOCIATE PROFESSOR OF INFORMATION
SCHOOL OF INFORMATION AND ASSOCIATE PROFESSOR OF PUBLIC BEHAVOR & HEALTH EDUCATION
SCHOOL OF PUBLIC HEALTH
UNIVERSITY OF MICHIGAN
ANN ARBOR, MI 48109-1285

VISWESWARAN, SHYAM, PHD, MD
ASSOCIATE PROFESSOR
DEPARTMENT OF BIOMEDICAL INFORMATICS
UNIVERSITY OF PITTSBURGH SCHOOL OF MEDICINE
PITTSBURGH, PA 15206

SCIENTIFIC REVIEW OFFICER

HUANG, ZOE E, MD
SCIENTIFIC REVIEW OFFICER
DIVISION OF EXTRAMURAL PROGRAMS
NATIONAL LIBRARY OF MEDICINE
NATIONAL INSTITUTES OF HEALTH
BETHESDA, MD 20892

EXTRAMURAL SUPPORT ASSISTANT

MCNAIR, JOSEPH G.
EXTRAMURAL SUPPORT
DIVISION OF EXTRAMURAL PROGRAMS
NATIONAL LIBRARY OF MEDICINE
NATIONAL INSTITUTES OF HEALTH
BETHESDA, MD 20892

PROGRAM REPRESENTATIVE

SIM, HUA-CHUAN, MD
CHIEF PROGRAM OFFICER
DIVISION OF EXTRAMURAL PROGRAMS
NATIONAL LIBRARY OF MEDICINE
NATIONAL INSTITUTES OF HEALTH
BETHESDA, MD 20892-7968

VANBIERLIET, ALAN, PHD
PROGRAM OFFICER
DIVISION OF EXTRAMURAL PROGRAMS
NATIONAL LIBRARY OF MEDICINE
NATIONAL INSTITUTES OF HEALTH
BETHESDA, MD 20892

YE, JANE, PHD
PROGRAM OFFICER
DIVISION OF EXTRAMURAL PROGRAMS
NATIONAL LIBRARY OF MEDICINE
NATIONAL INSTITUTES OF HEALTH
BETHESDA, MD 20892

OTHER REVIEW STAFF

NICOLAS, MINDY, BA
REVIEW PROGRAM SPECIALIST
DIVISION OF EXTRAMURAL PROGRAMS
NATIONAL LIBRARY OF MEDICINE
NATIONAL INSTITUTES OF HEALTH
BETHESDA, MD 20892-7968

OTHER

FLORANCE, VALERIE, PHD
DIRECTOR
DIVISION OF EXTRAMURAL PROGRAMS
NATIONAL LIBRARY OF MEDICINE
NATIONAL INSTITUTES OF HEALTH
BETHESDA, MD 20892-7968

Consultants are required to absent themselves from the room during the review of any application if their presence would constitute or appear to constitute a conflict of interest.
